# Relating Compulsivity and Impulsivity With Severity of Behavioral Addictions: A Dynamic Interpretation of Large-Scale Cross-Sectional Findings

**DOI:** 10.3389/fpsyt.2022.831992

**Published:** 2022-06-17

**Authors:** Zsolt Demetrovics, Wim van den Brink, Borbála Paksi, Zsolt Horváth, Aniko Maraz

**Affiliations:** ^1^Centre of Excellence in Responsible Gaming, University of Gibraltar, Gibraltar, Gibraltar; ^2^Institute of Psychology, ELTE Eötvös Loránd University, Budapest, Hungary; ^3^Amsterdam Institute of Addiction Research (AIAR), Academic Medical Center, University of Amsterdam, Amsterdam, Netherlands; ^4^Institute of Education, ELTE Eötvös Loránd University, Budapest, Hungary; ^5^Institut Für Psychologie, Humboldt-Universität zu Berlin, Berlin, Germany

**Keywords:** obsession-compulsion, addictive disorder, internet gaming disorder, neuroscientific theory of addiction, exercise dependence, compulsive buying, problematic gambling, work addiction

## Abstract

**Background and Aim:**

Impulsivity and compulsivity are two key temperament traits involved in behavior regulation. The aim of this study was to test several existing theories in explaining the role of impulsivity and compulsivity in symptom severity in various behavioral addictions.

**Methods:**

Data were collected from a (representative) general population sample (*N* = 2,710, mean age:39.8 years (SD:13.6), 51% woman), and from people who are at increased risk of having a behavioral addiction (*N* = 9,528 in total, mean age: 28.11 (SD:8.3), 34.3% woman), including people with problematic gaming and internet use, pathological gambling, exercise dependence, compulsive buying and work addiction. Symptom severity, reward driven impulsivity and relief driven compulsivity were assessed.

**Results:**

For non-problematic groups, impulsivity is present to about the same extent as compulsivity, whereas for problematic groups, compulsivity dominates over impulsivity in all groups (except for gambling). The strength of the correlation between impulsivity and compulsivity is higher in more severe forms of the disorders (from *r* = 0.18 to *r* = 0.59 in the representative population).

**Discussion:**

Based on these data, it appears that relief-driven behavior (negative reinforcement) dominates over reward-driven behavior (positive reinforcement) in more severe cases of a behavioral addiction.

**Conclusion:**

This is the first large-scale study to find empirical support for the neuroscientific theory on the dominance of compulsivity (“needing”) over impulsivity (“wanting”) in more severe cases of a behavioral addiction. Although longitudinal research is needed, a possible shift from impulsivity to compulsivity takes place, similar to substance use addictions, which maintains the circle of addiction.

## Introduction

Both impulsivity and compulsivity play an important role in the development and persistence of substance use disorders and behavioral addictions (1, 2). There are multiple theories regarding the dynamics of impulsivity and compulsivity and their role in the persistence and severity of addiction symptoms, however, further empirical research is needed to examine the role of impulsivity and compulsivity in addictive disorders (1–4). This study challenges several psychological and neurobiological theories on the role of impulsivity and compulsivity in the severity of behavioral addictions. Clarifying the role of impulsivity and compulsivity, two well-defined neurocognitive phenotypes, contributes to a better understanding of the etiology of behavioral addictions and to a comprehensive model that helps us to understand frequently occurring comorbidities (5, 6).

Behavioral addictions are a specific group of addictions that do not involve the use of a psychoactive substance (7, 8). Behavioral addictions can be conceptualized as excessive engagement in a specific behavior which contributes to functional impairment in the affected individual's life (e.g., negative impact on one's relationship with family or friends, on school or work performance, on mental and physical wellbeing). Moreover, behavior-specific compulsivity is also considered as a central element of addictive behaviors (2). DSM-5 only recognizes “gambling disorder” as an official behavioral addiction, but additionally includes “internet gaming disorder” as a condition that needs further study before it can be included in the DSM. Other behavioral addictions are loosely mentioned in the text of DSM-5 without providing any formal status and without explicit criteria, e.g., sex addiction, exercise addiction, and shopping addiction. ICD-11 includes both “gambling disorder” and “gaming disorder” under *Disorders due to addictive behaviors* within the higher-order category of *Disorders due to substance use or addictive behaviors* as the only two diagnosable conditions, while other behavioral addictions remain diagnosable under *Other specified impulse control disorders*. In addition to gambling disorder and gaming disorder, multiple excessive and problematic behavior tendencies are considered in the literature as potentially addictive behaviors, though they are not included specifically in the DSM-5 or the ICD-11 as addictive disorders (e.g., problematic internet and/or social media use, compulsive buying, hypersexuality, exercise addiction, work addiction, hair pulling disorder or trichotillomania) (7). Excessive and problematic forms of these behaviors can lead to similar symptoms and impairments like psychoactive substance-related addictions (e.g., salience or craving, coping motivations, tolerance, withdrawal symptoms, intra- and interpersonal conflicts, relapse) (2, 9). Apart from questions about the diagnostic status of some behavioral addictions, the field of behavioral addiction is plagued by uncertainties and concerns due to the lack of a comprehensive theoretical framework, especially in terms of etiology (7).

Impulsivity has been defined as a trait leading to unplanned but rewarding actions, which are unduly risky or inappropriate to the situation and often result in undesirable consequences (10, 11). Compulsivity, on the other hand, has been defined as loss of control over goal-directed behavior (12). Whereas impulsivity is driven by desire, pleasure, arousal and gratification, compulsivity is driven by the possibility to alleviate anxiety or discomfort (13). Both types of behavior, however, share the inability to inhibit or delay the repetitive behavior (14), as well as the lack of response inhibition, poor planning, and sensitivity to (positive or negative) reward expectation. Empirical evidence also confirms the need to treat impulsivity and compulsivity as qualitatively different constructs (15).

In addictive behaviors, impulsivity has been recognized as a relevant risk factor for (early) substance use initiation (e.g., the transition from the naïve condition to recreational use and from recreational use to abuse and dependence), for persistence of use (e.g., drugs may harm the cognitive systems that control behavior) and for relapse (e.g., suppressing behavioral control over positive reinforcing behaviors) (16, 17). Although not planned, impulsive behavior is driven by the promise of reward (positive reinforcement). The role of compulsivity in addictive behaviors is less clear. Some studies claim that at later stages of the addiction, compulsivity becomes an interaction that derives from need for relief from anxious, uncomfortable feelings: negative reinforcement (18). Oldham et al. (19) postulated an “addictive cycle” encompassing both impulsivity and compulsivity, including three stages: (1) binging (related to impulsivity), (2) preoccupation/anticipation, and (3) withdrawal/negative affect (related to compulsivity). This model has been successfully used to understand and treat alcohol addiction (20).

In line with these, numerous existing studies highlighted the possible risk role of impulsivity and compulsivity in various forms of behavioral addictions. Namely, higher levels of impulsivity and compulsivity were associated with increased rates of gaming disorder (21, 22) problematic internet use (23, 24), gambling disorder (25, 26), exercise addiction (27, 28), compulsive buying disorder (29, 30), work addiction (31, 32) and hair pulling disorder (33, 34). However, it is important to note that there might be differences between behavioral addictions in their associations with impulsivity and compulsivity (e.g., impulsivity might have a greater exploratory role in gaming disorder than compulsivity, whereas in gambling disorder both impulsivity and compulsivity might have a significant impact) (1, 35).

In the scientific literature, several theories have emerged, which have attempted to argue in favor or against pairing the constructs of impulsivity and compulsivity. Blum et al. (36) argue in favor of a pairing of both constructs. In their view, abnormal behaviors involving dopaminergic activity often reflect an insufficient usual feeling of satisfaction, a deficiency in the feeling of reward or a reward deficiency syndrome (RDS) (3, 37). The RDS is supported by neuroscientific findings, such as the sub-optimal functioning of the mid-brain resulting in dysfunction of dopamine receptors. The authors believe that reward deficiency is the core of addiction, and that the form of its manifestation (substance vs. non-substance related) is secondary. Dopamine sensitivity, which is strongly influenced by genetics, may mediate the following three mechanism, each involving impulsivity or compulsivity to a different extent: (a) the hedonic impact of reward (liking), (b) learned predictions about rewarding effects (learning), or (c) the pursuit of rewards by attributing incentive salience to reward-related stimuli (wanting) (38). The RDS model proposes that compulsivity and impulsivity are interdependent, and thus the level of compulsivity predicts the level of impulsivity and vice versa in behavioral addictions and higher scores of either or both are related to addiction severity.

Whereas the RDS postulates one single component as the underlying mechanism of addiction, the obsessive-compulsive disorder spectrum (OCDS) hypothesis (4, 39–41) proposes that many behaviors can be described as a result of two competing components: impulsivity and compulsivity, which are each governed by different neural mechanisms. Thus, problem behaviors differ by the (relative) amount of compulsivity and impulsivity that they incorporate (4). Behavior initiation (impulsivity) and behavioral inhibition (compulsivity) are considered temperament traits, thus strongly, biologically determined components of behavior regulation (39). The term *spectrum* refers to “a group of disorders that are presumed to be distinct from, but related to, OCD, and which are characterized by repetitive thoughts and/or behaviors” [(42) p.528]. According to this theory, impulsivity, a tendency toward rapid and unplanned behavior without regards to negative consequence, is triggered by risk seeking and the motivation to maximize pleasure (4, 10, 43). In contrast, compulsivity represents a tendency to perform repetitive acts to prevent or ameliorate negative consequence, thus compulsivity is driven by harm avoidance and anxiety-reduction (44, 45). The spectrum model proposes that compulsivity and impulsivity are largely independent and that the level of compulsivity does not predict the level of impulsivity in behavioral addictions and that severity is not related to either impulsivity or compulsivity.

For long, the dis-balance between brain circuits was believed to be the neurobiological background of addictive behaviors. A hyper-activated ventral affective circuit (which explains the increased anxiety and repetitive behaviors), and hypo-activated dorsal cognitive structures (explaining cognitive control deficits and inability to modulate emotional and behavioral responses) were considered as the core components of the addictive behavior (46). Later, two other circuits were added to the model: (1) the ventral cognitive circuit, which is connected to the limbic regions and thus involves emotions (47), and (2) the sensorimotor circuit, which plays a crucial role in the behavioral feedback loop (48). Neurodevelopmental studies based on substance addiction research suggest, that the brain continuously adapts to environmental factors, in this case to the addictive behavior, which changes the functionality of the brain circuit regulating the given behavior. The change in functionality is especially occurring during early stages of goal-directed behavior, which mediates the transition from goal-directed (impulsive) to habitual (compulsive) behaviors. The stage model postulates, that over the course of disease, a specific anatomical shift occurs, and, as a result, negative reinforcement (compulsivity) takes over positive reinforcement (impulsivity). Based on substance use research, the transition from recreational use to addiction involves neuroplasticity in brain structures (18, 49, 50). Neuroplasticity during the transition from recreational use to addiction is supported by converging neuroimaging research, which found evidence for the structural change in brain circuits (51, 52). Animal models also support the shift from impulsivity to compulsivity in the course of addiction. Highly impulsive rats (operationalized as a tendency to respond prematurely) are consistently found to (1) tolerate foot-shock better to receive cocaine and to (2) relapse after abstinence more often than their non-impulsive counterparts (53). In other words, the animal model of addiction postulates that high levels of impulsivity predispose the development and maintenance of compulsive drug-taking (54). This model proposes that compulsivity and impulsivity are largely interdependent and that the level of impulsivity predicts the level of compulsivity in behavioral addictions and that severity is mainly related to the level of compulsivity.

Impulsivity and compulsivity respond differently to medication. Compulsivity is sensitive to treatment with (high doses of) selective serotonin reuptake inhibitors (SSRIs), and this change is also associated with both structural and functional integrity in the orbitofrontal cortex (55, 56). Impulsivity (induced by d-amphetamine), on the other hand seems to react better to benzodiazepines, such as diazepam (57). Therefore, clarifying the role of impulsivity and compulsivity may enhance the treatment of addictive disorders considering severity.

The aim of the current study was, therefore, to clarify the relation between impulsivity and compulsivity in behavioral addictions (specifically: gaming disorder, problematic internet use, pathological gambling, exercise dependence, compulsive buying, work addiction, hair pulling) and the relation of these traits to symptom severity using similar self-report data on impulsivity and compulsivity in a general population sample and in a series of populations with different behavioral addictions, including people with addictive internet gaming, gambling, physical exercise, hair pulling, and buying/shopping. These potentially addictive behaviors were selected for the analyses as there were attempts in the literature to conceptualize them as addictive behaviors (58–64), they can share similar symptomatic characteristics and features (e.g., salience/craving, mood modification motivations) (2, 9), and theoretical models of addictive disorders also highlighted their role (e.g., hair pulling/trichotillomania, compulsive buying, problematic gambling were included in the OCSD model) (4). Consistent with the existing neurocognitive theories and animal models supporting the dynamic shift from impulsivity to compulsivity, we hypothesized that as severity of addiction symptoms increases, compulsivity will progressively dominate over impulsivity. At the same time, it is expected that the correlation between impulsivity and compulsivity increases with increasing symptom severity, as predicted by the cycle of addiction.

## Methods

### Data Collection

The protocols for the general population and the addictive behavior samples were approved by the Institutional Review Board of ELTE Eötvös Loránd University in Budapest, Hungary. Data were collected in accordance with the Declaration of Helsinki. Participants provided written consent before starting to fill out the questionnaires.

Several samples were collected: a nationally representative general population sample of 2,710 participants and several specific samples (*N* = 9,696 in total) with participants meeting threshold criteria for behavioral addiction (problematic gaming, problematic gambling, physical exercise dependence and compulsive buying). Details about each sample and data collection procedures are provided below.

#### Representative General Population

Symptoms of behavioral addictions were assessed within the framework of the National Survey on Addiction Problems in Hungary (NSAPH), i.e. a representative sample of the general population of Hungary (65). In this survey, both chemical addictions (i.e., tobacco smoking, alcohol and other substances) and various behavioral addictions (i.e., pathological gambling, internet addiction, compulsive buying, eating disorders, work addiction, physical exercise dependence) were assessed.

The target population of the survey was the total population of Hungary between 18 and 64 years (6,703,854 persons). The sampling frame consisted of the whole resident population with a valid address according to the register of the Hungarian Central Office for Administrative and Electronic Public Services. Data collection took place in a gross sample of 3,183 individuals, stratified according to geographical location, degree of urbanization, and age (overall 186 strata) representative of the sampling frame. Participants were surveyed with so-called “mixed methods” via personal visits. Questions on background variables and introductory questions referring to specific disorders were asked during face-to-face interviews, while symptom scales were self-administered as paper-and-pencil questionnaires. These questionnaires were returned to the interviewer in a closed envelope to ensure confidentiality. Participants were informed both verbally and in a written form that participation in the study was voluntary and anonymous. The net sample size was 2,710 (response rate: 85.1%). Those who reported one or more of the following activities were asked to proceed with the questionnaire measuring the related behavioral addiction: physical exercise or buying at least once a week, working at least 40 h a week, or ever gambled.

#### Specific Populations

Specific populations were recruited following a similar protocol. Participants were approached at random times online, or during the opening hours in the given venue (e.g., shopping mall, gambling venue, fitness center). Inclusion criteria were: (a) at least 18 years old, and (b) pursuing the specific activity (e.g., shopping) at least once a week. After providing written informed consent, participants could begin the questionnaire (online) or were sent an e-mail containing the link to enter the online questionnaire. Those who participated did not receive any financial compensation for participating (except when mentioned otherwise); however, they did receive a brief feedback. Sample-specific processes are described below.

#### Internet Gaming

Data collection took place online with the cooperation of a popular Hungarian gaming magazine (GameStar) targeting the entire gamer community in Hungary including both PC and console gamers (66). A participation call was posted online via the magazine's website and Facebook page three times from August to September 2014. Participants provided informed consent by ticking a box if they agreed to continue and participate in the study. Although parental consent was sought for participants below age 18, they were later excluded from the sample of the current analysis. A shopping voucher of 90,000 Hungarian forints (HUF) (approx. 300€) was drawn between participants that fully completed the survey. A total of 7,757 gamers started the survey. After excluding participants with too many missing data or inconsistencies, 4,751 gamers (61.2%) remained, of which 3,226 were above age 18.

#### Gambling

Data was gathered from nine gambling venues (on 33 occasions) and 26 lotteries (on 115 occasions) in and around Budapest (67). A total of 1,035 individuals were sent the link to the online questionnaire by e-mail. Of these, 533 provided valid answers. In addition to the online call, we provided the possibility for offline responding during recruitment and this resulted in an additional 254 answers resulting in a final sample size of 586 participants (37.4%) with valid answers on the total scale of the South Oaks Gambling Screen (SOGS). Those who had missing data on the latter questionnaire were excluded from the analyses.

#### Exercise

Participants were recruited from one semi-professional club for triathlon training and 17 different sports centers representative for Budapest (68, 69). University students assisted with data collection, which resulted in 4,589 people signing the informed consent and they were all sent the study link. Of these, 2,744 (59.8%) had valid answers on the total scale of the Exercise Dependence Scale (EDS) and were included in the current study. Those who had missing data on the latter questionnaire were excluded from the analyses.

#### Compulsive Buying

Our target population was customers from three different shopping malls in Budapest and one in another town in Hungary (Gyor). Overall, 5,068 people met inclusion criteria, provided written consent and their e-mail address. An e-mail was sent to this address containing the study link and password within 24 hours of providing consent (29, 70). Overall, 1,776 individuals started with the questionnaire and 1,447 (28.6%) of them completed and provided valid responses with no missing data on the Questionnaire About Buying Behavior (QABB). Those who had missing data on the latter questionnaire were excluded from the analyses.

#### Hair-Pulling

Data were collected in an online survey that was advertised as “Win three tickets for the Sziget Festival with your habits” on Hungarian general news and magazine websites. Participants were entered into a price drawing where three incentives (valued at €900) were offered. Participants could reach the survey between January and August 2014. Only individuals older than 18 years could take part. In total, 4,177 people participated in the online questionnaire, of which 822 reported to have pulled their hair at least once (i.e., 1679 individuals were excluded as they reported to not pulling ever their hair) and provided valid responses on the Massachusetts General Hospital Hair-pulling Scale (MGH-HPS) (34). Their data were used for further analyses.

### Measurement

#### Behavioral Addictions

*Gaming*. The 10-item Internet Gaming Disorder Test (IGDT, 65) was used to assess Internet gaming symptoms. The instrument was designed to reflect the DSM-5 criteria for internet gaming disorder, via items such as “Have you ever in the past 12 months unsuccessfully tried to reduce the time spent on gaming?”. Response options for the ten items were “never”, “sometimes”, and “often”. The IGDT-10 items were then dichotomized into a “yes” (1, “sometimes” and “often”) and “no” (0, “never”) to resemble the dichotomous structure of the DSM-5 criteria. Given that questions 9 and 10 are related to the same criterion, they were combined during scoring, that is, answering “often” on either item 9 or item 10 (or both items) scored only 1 point. Based on latent class analysis, the endorsement of 5 or more points was considered problematic Internet gaming (66). Cronbach alpha was 0.79 (specific sample).

#### Internet Use

The 18-item Problematic Internet use questionnaire (71) was created based on Young's Internet Addiction Test (72). A sample item is “How often do you neglect household chores to spend more time online?”. Response alternatives were between 1 (never) and 5 (always). Test–retest correlation of the PIUQ was 0.90. Based on latent class analysis, a cut-off value of 41 was suggested to separate problematic and non-problematic users (71). Cronbach alpha was 0.91 (representative sample).

#### Pathological Gambling

The South Oaks Gambling Screen [SOGS, (73, 74)] was used to assess symptoms of pathological gambling. Based on the DSM-III criteria of pathological gambling (e.g., “Did you ever gamble more than you intended to?”), the instrument contains 20 items, where items are scored yes (1) or no (0). A total score of 0 indicates the absence of problems, 1–2 indicates some problems, scores 3 to 4 refer to mild problems, and a score of 5 or more indicates the probable presence of pathological gambling. Cronbach alpha was acceptable in the representative and in the specific sample as well (both 0.80).

#### Exercise Dependence

We used the 21-item Exercise Dependence Scale Revised version [EDS (75), 74, (76)] to assess the severity of exercise dependence symptoms (e.g., “I am unable to reduce how intense I exercise”). Risk of exercise dependence was defined as scoring 5–6 on the 6-point Likert scale on at least three of the seven DSM-IV dependence criteria, non-dependent symptomatic individuals scored 3–4 on the Likert scale on at least three DSM-IV dependence criteria or had scores 5–6 combined with scores 3–4 on three DSM-IV dependence criteria, but not meeting the at-risk condition. Finally, individuals who scored 1 or 2 on the Likert scale on at least three criteria but did not meet the conditions of the non-dependent symptomatic group were classified as non-dependent asymptomatic. Scale reliability was 0.92 in the representative and 0.90 in the specific sample.

#### Compulsive Buying

The Questionnaire About Buying Behavior [QABB (29, 77, 78)] was used to identify compulsive buyers (e.g., “Have you ever asked someone to go shopping with you so you wouldn't spend too much?”). The 19 dichotomous items assess spending, shopping and affects. Individuals scoring 8 or more are classified as problematic buyers. Cronbach alpha was acceptable: 0.85 in the representative and 0.73 in the specific sample.

####  Work Addiction

The symptom severity of work addiction was assessed with the Work Addiction Risk Test (WART) (79, 80). Only those were asked to fill out the questionnaire who worked at least 40 h a week. Respondents read the 17 items which describe work habits and mark a 4-point scale anchored by “always true” and “never true” (e.g., “19. It is hard for me to relax when I'm not working”). The WART has excellent test-retest reliability and concurrent validity. Based on latent profile and sensitivity analysis, participants scoring 51 or more (out of the possible 64) are classified as problematic (81). Cronbach alpha was excellent (0.89, representative sample).

#### Hair-Pulling

Hair-pulling was assessed by the Massachusetts General Hospital Hair-pulling Scale (MGH-HPS). This scale is based on the Yale–Brown Obsessive–Compulsive Scale [Y-BOCS, (82)]. The MGH-HPS has previously demonstrated strong test–retest reliability (r = 0.97) (83). The questionnaire is self-administered and contains items like “On an average day, how often did you feel the urge to pull your hair?” Participants rate severity, urge to pull, actual pulling, perceived control and associated distress from 0 (no symptom) to 4 (extremely strong symptom). The questionnaire provides an estimate of symptom severity in the past seven days. No valid grouping (i.e., pathological vs. non-pathological hair pullers) has been developed for this instrument yet. Internal consistency was high in the current sample (α = 0.92).

#### Impulsivity and Compulsivity

##### Impulsivity

The short, 21-item version of the Barratt Impulsiveness Scale (84) was used to assess impulsivity [BIS, (85)]. In this short version of the BIS a new factor structure was developed based on the initial items, covering cognitive impulsivity, behavioral impulsivity and impatience/restlessness and confirmed in two independent samples. Items are evaluated between 1 (never) and 4 (very often/always) with total scores ranging from 21 to 84. Cronbach's alpha was acceptable in the representative (0.70) and good in the specific samples (gaming: 0.80, gambling: 0.80, exercise: 0.77, buying: 0.82).

##### Compulsivity

Compulsivity was measured with the Obsessive-compulsive subscale derived from the Symptom Check List [SCL-90, (86, 87)] in the representative study (9 items) and from its short version, the Brief Symptom Inventory [BSI, (88)] in the specific samples (6 items). The SCL-90 is a 90-item, and the BSI is a 53-item self-report symptom inventory designed to reflect psychiatric symptom patterns of psychiatric and medical patients. In both versions, each item was rated on a five-point scale of distress from 0 (not at all) to 4 (very much). Therefore, the Obsessive-compulsive subscale scoring ranged between 0 and 36 for the representative, and between 0 and 24 for the specific samples. Cronbach alpha was good in the representative (0.87) and in the specific samples (gaming: 0.78, gambling: 0.78, exercise: 0.77, buying: 0.78).

### Data Analysis

Impulsivity and compulsivity were standardized within each sample to minimize bias between the different samples. Standardization involved subtraction of the mean of all data points from each individual data point divided by the standard deviation of all data points. *T*-tests (for two groups) and F-tests (for more than two groups) were used to assess group differences on impulsivity and compulsivity, and Tukey's test was applied to explore *post-hoc* group differences in F-tests. Pearson correlation was used to assess the association between impulsivity and compulsivity. Accounting for overlap between variables, the effect was tested using linear regression analysis using least-squares. Symptom severity scores of behavioral addictions was entered as the dependent variable, whereas impulsivity and compulsivity were predictors. F values and corresponding significance levels indicate the goodness of model fit, whereas standardized betas for main effect and interaction (and corresponding significance level) will indicate the predictive potential of each independent variable. Cases with missing data were deleted pairwise.

Data were analyzed and visualized in R (89) using ggplot2 (90). All data and scripts are available open access on the following link: http://osf.io/q965k.

## Results

### Sample Description

In the representative sample (*N* = 2,710), prevalence of the various behavioral addiction varied between 0.2% (exercise) and 1.8% (internet use) in the entire sample, whereas the prevalence varied between 1.3% (gambling) and 15% (buying) among those who reported pursuing the given activity at least once a week. The proportion of participants with a behavioral addiction in the specific samples varied between 4.3% (exercise) and 13.3% (for both gambling and buying). Sex and age differed across samples: women were more likely to shop, and men were more likely to gamble or work (see Table 1). Mean age was slightly higher in the representative than in the specific populations (see Table 1).

**Table 1:** Sample characteristics.

**Study**	***N***	**% of women**	**% of men**	**Mean age (SD)**
Representative sample (total)^a^	2,710	51	49	39.8 (13.6)
Gambling				
Never gambled weekly	1,589	61.9	55.3	38.8 (13.8)
Non-problematic	827	31.0	30.0	41.6 (13.1)
Minor problems	204	5.9	9.2	41.5 (13.5)
Problematic	51	0.7	3.2	40.2 (14.8)
Pathological	39	0.5	2.4	34.2 (11.4)
Internet use				
Less than once a week	1,744	65.1	63.6	43.2 (13.3)
Non-problematic	918	33.5	34.3	34.0 (11.8)
Problematic	48	1.4	2.1	26.7 (10.4)
Work				
Less than 40h/week	1,403	59.2	44.1	40.6 (15.7)
Non-problematic	1,265	39.4	54.2	38.8 (10.9)
Problematic	42	1.4	1.7	42.6 (10.5)
Exercise				
Less than once a week	2,252	85.5	80.6	41.4 (13.4)
Asymptomatic	268	8.8	11.0	32.8 (12.1)
Symptomatic	182	5.5	8.0	30.1 (11.1)
At-risk	8	0.1	0.5	33.8 (18.6)
Buying				
Less than once a week	2,517	90.6	95.3	40.0 (13.5)
Non-problematic	164	7.6	4.4	37.9 (14.5)
Problematic	29	1.8	0.3	32.4 (10.9)
Specific samples				
Gaming (total)	3,600	7.4	92.5	24.3 (5.8)
Non-problematic	3,502	96.3	97.4	24.3 (5.8)
Problematic	98	3.7	2.6	23.4 (5.7)
Gambling (total)	586	38.4	61.6	38.2 (13.8)
Non-problematic	270	34.3	64.9	40.1 (12.9)
Minor problems	163	30.5	23.6	39.5 (14.8)
Problematic	75	15.8	8.0	38.9 (14.9)
Pathological	78	19.4	3.6	35.7 (13.1)
Exercise (total)	2,744	56.5	43.5	34.5 (8.5)
Asymptomatic	674	25.4	23.5	36.1 (8.8)
Symptomatic	1,951	70.0	72.5	34.0 (8.3)
At-risk	119	4.6	4.0	32.0 (7.8)
Buying (total)	1,447	62.6	37.4	31.1 (12.1)
Non-problematic	1,254	85.1	89.1	31.3 (12.2)
Problematic	193	14.8	10.9	30.4 (11.5)
Hair-pulling (total)	822	63.3	36.7	28.5 (8.0)

a *Only those, who indicated pursuing the given activity (i.e. gambling) at least once a week were asked to fill out the questionnaire from the total sample (N = 2710) to measure problematic behavior*.

### Severity, Impulsivity and Compulsivity

As expected, problem behaviors were more severe in the specific samples than in the representative sample (Table 2). As a general trend, as behavior pathology emerges and symptom severity increases, so do impulsivity and compulsivity in the representative and in most of the specific samples, and most group differences between the problematic groups, and problematic vs. non-problematic groups are significant (see Table 2).

**Table 2:** Comparison of impulsivity and compulsivity according to severity groups.

**Study**	**Compulsivity std (SD)**	**Compulsivity raw (SD)^**b**^**	**F/t**	**Group difference**	**Impulsivity std (SD)**	**Impulsivity raw (SD)**	**F/t**	**Group difference**	**r (impulsivity, compulsivity)**
Representative sample (total)^a^		12.6 (6.22)				39.3 (7.7)			0.27*
Gambling									
Non-problematic (a)	−0.06 (0.93)	12.2 (5.8)	10.4*	a < c*	−0.16 (0.89)	38.1 (6.8)	17.7*	a < c^+^	0.18*
Minor problems (b)	0.005 (0.93)	12.6 (5.8)		a < d*	0.03 (0.90)	39.6 (6.9)		a < d*	0.22*
Problematic (c)	0.52 (0.99)	15.8 (6.1)		b < c^+^	0.28 (0.75)	41.5 (5.7)		c < d^+^	0.07
Pathological (d)	0.78 (1.31)	17.4 (8.2)		b < d*	1.07 (1.01)	47.5 (7.7)		b < d*	0.54*
Internet use									
Non–problematic	−0.10 (0.81)	11.9 (5.0)	93.8*		−0.19 (0.92)	37.9 (7.1)	65.4*		0.28*
Problematic	1.08 (1.01)	19.3 (6.3)			0.94 (0.94)	46.5 (7.2)			0.59*
Work									
Non-problematic (a)	−0.12 (0.83)	11.8 (5.1)	0.01		−0.14 (0.95)	38.2 (7.3)	0.86		0.25*
Problematic (b)	−0.10 (0.96)	11.9 (6.0)			−0.28 (0.97)	37.1 (7.4)			0.41*
Exercise									
Asymptomatic (a)	−0.17 (0.81)	11.5 (5.0)	14.7*	a < b*	−0.18 (0.98)	37.9 (7.5)	5.2*		0.20^+^
Symptomatic (b)	0.18 (0.97)	13.6 (6.1)		a < c*	0.09 (0.94)	40.0 (7.2)		a < b+	0.24^+^
At-risk (c)	1.16 (1.74)	19.8 (10.8)		b < c^+^	0.42 (0.99)	42.5 (7.6)			0.41
Buying									
Non-pathological	−0.01 (0.88)	12.5 (5.5)	4.8^+^		0.15 (1.01)	40.5 (7.8)	0.08		0.24^+^
Pathological	0.38 (0.84)	14.9 (5.2)			0.21 (0.97)	40.9 (7.4)			0.37^+^
Specific samples									
Gaming (total)		4.56 (4.4)				38.9 (7.6)			0.32*
Non-problematic	−0.04 (0.95)	4.41 (4.2)	240.7*		−0.02 (0.99)	38.7 (7.5)	55.4*		0.31*
Problematic	1.50 (1.40)	11.45 (6.17)			0.76 (1.14)	44.7 (8.7)			0.09^+^
Exercise (total)		3.99 (3.9)				48.8 (5.2)			0.19*
Asymptomatic (a)	0.07 (1.01)	4.25 (3.95)	38.5*	a > b*	0.03 (0.96)	49.0 (5.0)	29.2*	A > b*	0.14*
Symptomatic (b)	−0.27 (0.85)	2.93 (3.33)		a < c*	−0.20 (0.98)	49.0 (5.0)		a < c*	0.16*
At-risk (c)	0.50 (1.33)	5.69 (5.21)		b < c*	0.50 (1.38)	51.4 (7.2)		b < c*	0.34*
Gambling (total)		3.13 (3.76)				39.9 (7.44)			0.37*
Non-problematic (a)	−0.18 (0.87)	2.47 (3.41)	9.2*	a < d*	−0.29 (0.92)	37.7 (6.8)	25.4*	a < c*	0.35*
Minor problems (b)	0.02 (0.95)	0.01 (3.72)		b < d^+^	0.004 (0.93)	39.9 (7.0)		a < d*	0.28*
Problematic (c)	0.16 (0.99)	3.80 (3.72)			0.24 (1.04)	41.7 (7.7)		b < d*	0.29^+^
Pathological (d)	0.45 (1.29)	4.92 (5.03)			0.78 (0.91)	45.7 (6.7)		c < d^+^	0.38*
Buying (total)		4.22 (4.27)				39.7 (7.5)			0.33*
non-problematic	−0.10 (0.92)	3.80 (3.95)	93.2*		−0.10 (0.97)	38.9 (7.3)	105.7***		0.29*
Problematic	0.63 (1.23)	6.90 (5.24)			0.67 (0.96)	44.8 (7.2)			0.26*
Hair-pulling (total)		6.97 (5.31)				48.3 (36.6)			0.86*

std, impulsivity and compulsivity scores were standardized within samples; row, non-standardized scores. ^a^Only those, who indicated pursuing the given activity (i.e. gambling) at least once a week were asked to fill out the questionnaire from the total sample (N = 2,710) to measure problematic behavior. ^b^Compulsivity scores ranged between 0 and 36 in the representative, and between 0 and 24 in the specific samples. *p <0.001; ^+^p <0.01.

**Figure 1:**
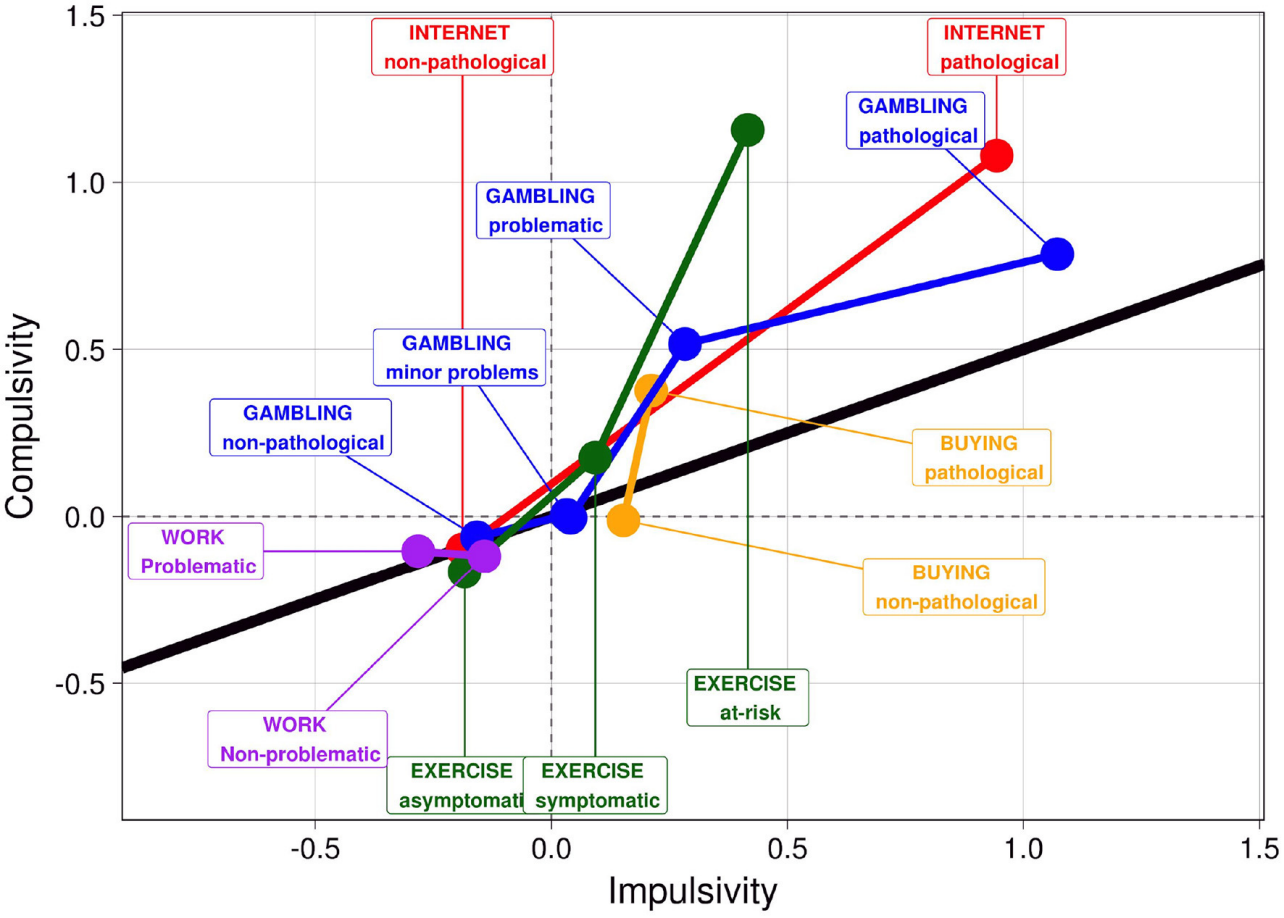
Impulsivity and compulsivity within problem groups in the representative sample. Impulsivity and compulsivity scores were standardized within the sample, and the standardized values are present in the Figure. The solid black line indicates the (theoretical) position when compulsivity and impulsivity are equally present in the given group. Values above the solid line indicate the dominance of compulsivity, whereas group means below the line indicate the dominance of impulsivity.

**Figure 2:**
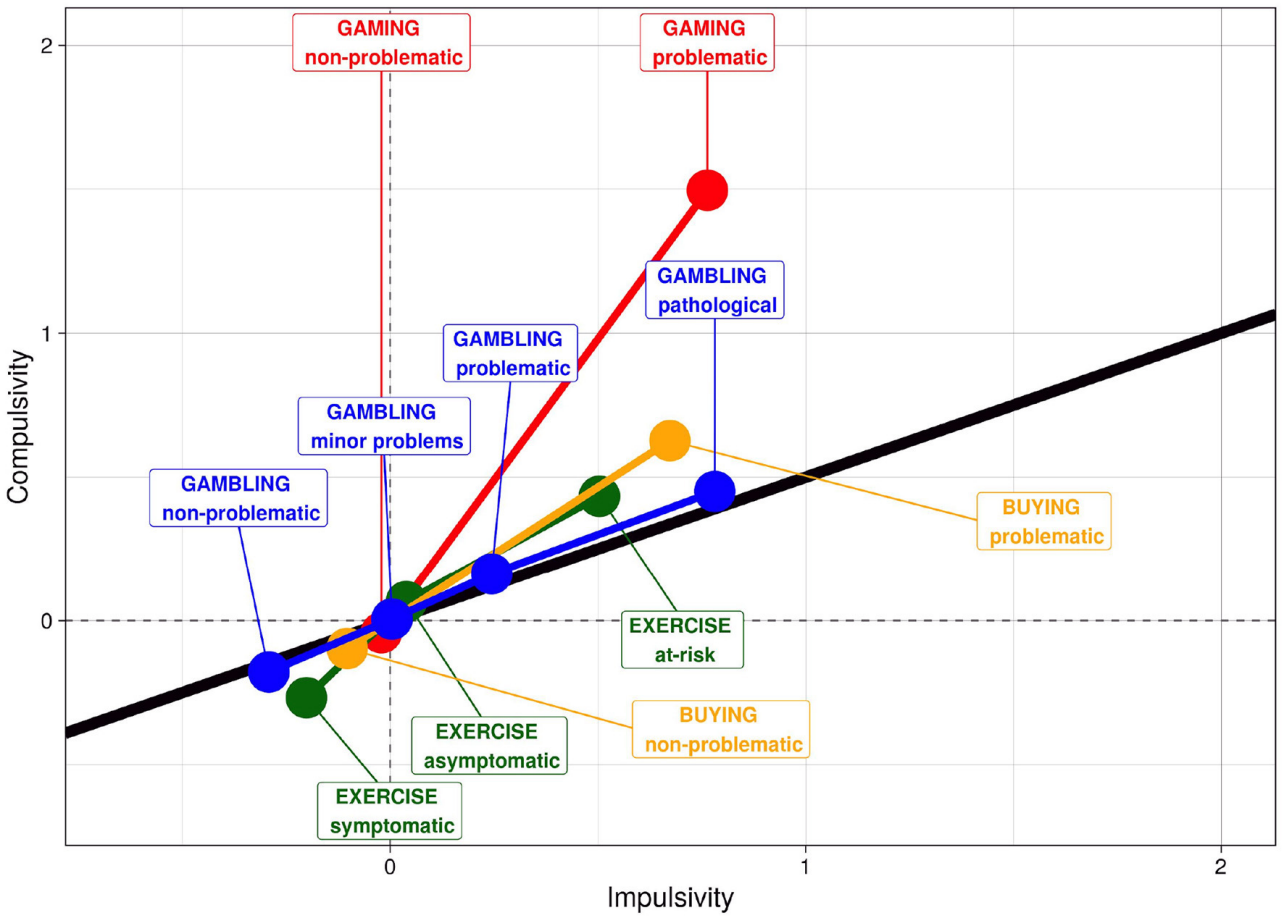
Impulsivity and compulsivity within problem groups in specific samples. Impulsivity and compulsivity scores were standardized within the sample, and the standardized values are present in the Figure. The solid black line indicates the (theoretical) position when compulsivity and impulsivity are equally present in the given group. Values above the solid line indicate the dominance of compulsivity, whereas group means below the line indicate the dominance of impulsivity.

Regarding the correlation between impulsivity and compulsivity, a clear trend of increasing strength of the correlation can be seen as behavior pathology emerges and becomes more problematic. This is true for almost all groups in the representative sample. Figure 1 (and Appendix 1) shows that as symptom severity increases, compulsivity increases faster than impulsivity in the representative sample. This is especially evident in compulsive buying, exercise addiction and internet addiction, and to a lesser extent in gambling addiction in the representative sample. The trend is less clear in work addiction, although there is a non-significant difference between the groups on impulsivity and compulsivity. The most impulsive problematic groups are pathological gamblers and internet users. Compulsivity is highest in at-risk exercisers, and to a lesser extent, pathological internet users. Figure 2 (and Appendix 2) shows that this relationship of behavioral symptom severity with impulsivity and compulsivity is less evident in the specific samples. As symptom severity increases, so do impulsivity and compulsivity, but within most problem behaviors compulsivity is increasing faster than impulsivity, especially in problematic gaming. The increase in impulsivity and compulsivity is approximately equal in gambling and buying. Like the representative sample, problematic gamers report the highest level of impulsivity (and compulsivity). In general, the correlation between compulsivity and impulsivity in the non-problematic groups in the specific samples is higher than in non-problematic groups within the representative sample, indicating the presence of possible floor effects (see Table 2).

Impulsivity, compulsivity, and their interaction were entered in the same linear regression model with symptom severity as the dependent variable. The model was significant in three out of four behavioral addictions in the representative sample (work addiction: F = 24.16, *p* <0.001, exercise: F = 12.24, *p* <0.001; gambling: F = 36.52, *p* <0.001), but not in buying (F = 2.01, *p* = 0.11). In the significant models, impulsivity and compulsivity were independently significant predictors of symptom severity (work: β_imp_ = 0.24, β_comp_ = 0.29, both *p* <0.001; exercise: β_imp_ = 0.82, *p* <0.05, β_comp_=1.02, *p* <0.001, gambling: β_imp_ = 0.05, *p* <0.001, β_comp_ = 0.056, *p* <0.01), whereas the interaction term was only significant in gambling (*p* <0.001), but not in work (*p* = 0.33) or exercise (*p* = 0.93).

Impulsivity, compulsivity, and their interaction also significantly predicted symptom severity in all specific populations (buying: F = 129.1, *p* <0.001; exercise: F = 60.78, *p* <0.001; gambling: F = 32.24, *p* <0.001; gaming: F = 272.2, *p* <0.001; grooming: F = 4.72, *p* <0.01). In buying, impulsivity and compulsivity were significant (β_imp_ = 0.013, *p* <0.05, β_comp_ = 0.09, *p* <0.01), but their interaction was not (*p* = 0.33). In exercise, impulsivity was significant (β = 0.08, *p* <0.001), but compulsivity was not (*p* = 0.88) and neither was the interaction between the two (*p* = 0.17). Impulsivity (β = 0.02, *p* <0.001), compulsivity (β = 0.18, *p* <0.05), as well as their interaction (β = 0.004, *p* <0.001) independently predicted the symptom severity of exercise, although the effects were small and similar to those of gaming (β_imp_ = 0.012, *p* <0.01; β_comp_ = 0.06, *p* <0.01; β_interaction_ = 0.0013, *p* <0.05). None of the effects or the interaction was significant in grooming (all *p* > 0.13).

## Discussion

Neurocognitive theories and animal models hypothesize a dynamic shift from high impulsivity to even higher compulsivity with the emergence of and with increasing severity of chemical and behavioral addictions. Although this hypothesis has gained extensive support in animal studies on substance use disorders (54), this is the first study to provide empirical human evidence for behavioral addictions. With higher levels of symptom severity of behavioral addictions, compulsivity was a stronger predictor of symptom severity than impulsivity, whereas the level of both traits is higher in higher levels of symptom severity than in lower levels of symptom severity of behavioral addictions. Furthermore, the correlation between these traits becomes stronger when behavioral addiction symptoms become more pronounced. This suggests that during the course of addiction the problem behavior becomes more and more compulsivity driven. As proposed by the stage model (18, 49, 50), this change may represent brain adaptations to environmental changes resulting in a change from goal-directed (reward driven) behavior to habitual behavior, which is similar to the dynamics assumed for the course of chemical addictions (substance use disorders).

The role of compulsivity in the development of substance-related addictions has long been established. For example, high impulsivity predicts compulsive cocaine taking in rats (91). Although higher reactivity to novelty predicted early vulnerability to cocaine use, this tendency did not determine the progression to addiction, only compulsivity did. This mechanism is interpreted as a failure in top-down executive control over maladaptive habit learning, and a shift from ventral to the dorsal striatum pathology (52, 92). Our findings indicate that the shift from impulsivity to compulsivity occurs independent of the used substance, but instead is the result of repeated rewarding behaviors and or the repeated negative consequences of it. Although the dynamics in the development of behavioral addictions are often assumed to be similar to chemical addictions, this is the first study to provide empirical support for this assumption in a large-scale survey.

Our finding, that with increasing symptom severity of the pathological behavior compulsivity becomes more pronounced than impulsivity, suggests that in later stages or in more severe forms of the pathological behavior, the motivation to *avoid* or resolve unpleasant feelings is driving the persistence of the pathological behavior instead of seeking reward. This, and the mechanism of operant conditioning (as well as modifications in brain structures) create a vicious circle of addiction, in line with previous theories [e.g., (19, 50, 54)]. Our findings do not support the Reward Deficiency Syndrome theory (36), which suggests that reward-seeking behavior motivates addiction in most if not all stages of the addictive circle. The current evidence suggests that the more advanced a behavioral addiction is, the more compulsivity dominates over impulsivity, and not vice versa.

Pathological gambling was the only behavioral addiction where impulsivity and compulsivity were elevated to about the same extent with increasing symptom severity in both samples. Until recently, pathological gambling was generally considered an impulse-control disorder given the consistent association between gambling and impulsivity (25, 93–95). Moreover, in line with the present findings, previous studies showed positive associations between compulsivity-related cognitive deficits and gambling disorder (26). In (pathological) gambling, the rewarding value of the behavior appears to be just as important as the habituation even in more advanced or more severe cases. This is further supported by the increasing strength of the correlation between impulsivity and compulsivity among problem gamblers in the representative sample. These findings are consistent with the obsessive-compulsive spectrum disorder hypothesis (40). The question as to whether impulsivity is a biologically determined risk factor or a consequence of the problem behavior remains unanswered, although there is evidence, that impulsivity is likely to precede the onset of problematic gambling rather than to develop as a consequence (96). Moreover, the highlighted role of impulsivity on pathological gambling can also be accounted for impulsive, gambling-specific behaviors and cognitions, such as chasing losses, premature and non-planned decision making, irrational beliefs, risk assessment (97). These impulsive, behavior-specific deficits might have a greater role in gambling disorder (the only behavioral addiction that is considered by both the DSM-5 and the ICD-11) compared with other, behavioral addictions.

In Internet use or problematic gaming (currently listed under the category of “internet gaming disorder” in DSM-5), compulsivity is more elevated than impulsivity in the more severe cases of the disorder. That is, the present findings are line with previous literature data which showed positive link between gaming disorder and obsessive-compulsivity (21). Previous studies report varying, small-to-large associations between problematic internet use and obsessive-compulsive symptoms [see Carli et al. (98)] and medium correlations between internet use and impulsivity (99). It should be noted, however, that the strength of the correlation is elevated at greater symptom severity in the representative sample and decreased in the specific population among people displaying more severe symptoms. This latter effect is likely to be due to the young age of these participants (lower than in the representative sample). Further studies are needed to generalize the results to a larger population. Furthermore, given the substantial difference between non-problematic and problematic internet users and gamers in terms of compulsivity and impulsivity, future studies should be careful in selecting the sample in terms of severity when assessing impulsivity or compulsivity.

In compulsive buying the general trend is in line with the main findings: compulsivity is more pronounced than impulsivity in more severe cases, although this effect is stronger in the representative than in the specific sample. Several studies found a medium-strength linear relationship between at least some aspect of impulsivity and the severity of compulsive buying (100–102) and a slightly weaker relationship between compulsivity and compulsive buying [e.g., (103)], which is in contrast with the current findings. In addition to these, other studies reported significant, positive and similar levels of correlations (moderate) between compulsive buying and impulsivity and obsessive-compulsivity (29).

One of the most compulsive groups in the representative study was the group of exercisers at-risk of addiction, although this trend was less prominent in the specific sample. Exercise is considered a heterogeneous activity, but the common reason is often to feel “in shape”, and look good, and this is the anticipated reward. However, exercise can also be seen as self-punishing behavior when physical effort and willpower are taken to the extreme (58). Not surprisingly, for these reasons, exercise addiction is primarily considered a disorder related to obsession-compulsion (104, 105). In line with this, some previous studies reported positive associations between exercise addiction and obsessive compulsivity (28).

Work addiction was only tested in the representative sample. Here, the trend of the compulsivity being more dominant than impulsivity in the more severe forms of the disorder is less clear than in the other behavioral addictions. Although the correlation between impulsivity and compulsivity is substantially higher in the problematic than in the non-problematic group, group differences on these variables are non-significant. Contrary to this finding, some literature data suggested positive relationships between work addiction and impulsivity and obsessive-compulsivity (31, 32). Further studies should select an assessment instrument that is better at predicting work addiction (i.e., validated with a clinical diagnosis). Nevertheless, the data clearly indicates that the association between impulsivity and compulsivity strengthens at more severe stages, therefore it is likely to play a role in forming a “vicious circle” in symptom maintenance.

Although grouping according to symptom severity was not available for hair-pulling (trichotillomania), the association between impulsivity and compulsivity is remarkably strong. Trichotillomania has previously been associated to impaired motor inhibition (44) and to other forms of impulse-control deficits (33, 106) in a way similar to patients with obsessive-compulsive disorder. Therefore, our finding of a strong correlation between impulsivity and compulsivity needs to be replicated.

## Limitations and Future Directions

The findings of the current study are limited by the cross-sectional nature of the data. It was not possible to determine the progression between different stages of behavioral addictions (e.g., progression from a more impulsive stage to a more compulsive stage), as well as how the presence of more severe symptom severity can account for the temporal progression of the different stages of behavioral addictions. Future prospective studies should explore whether differences in impulsivity and compulsivity precede the development of the disorders or develop as a consequence of the worsening of the disorder, and whether these differences account for the presence of comorbid disorders, such as attention deficit and hyperactivity disorder (107). In addition, future studies should use experimental designs, including the use of reward devaluation to assess the development of habitual behaviors [cf. (52, 91)]. Furthermore, impulsivity and compulsivity may themselves not be unitary (100), and could be further fractionated, especially since the reliability of the obsessive-compulsive scale of the SCL-90 is debated (108). Moreover, the comparison of the effects of impulsivity and obsessive-compulsivity is limited due to the disparate measurements of these constructs. Obsessive-compulsivity was measured as state-level psychopathological symptoms, whereas impulsivity was conceptualized as a personality trait. However, in order to limit the length of the questionnaire, the applied measurements of obsessive-compulsivity only provided superficial assessments of the construct, and the use of a more detailed and comprehensive symptom checklist would have been desirable. The elapsed time between the administration of the representative data collection (2006) and the last specific data collection (2015) is perhaps too long to treat data from the different samples as homogenous, because of technical changes during this period (i.e., problematic internet use and gaming). Moreover, it should be noted that this study used self-reported measures of impulsivity as a personality trait and compulsivity as psychopathological state symptoms rather than as specific aspects of the addictive behavior per se, whereas most animal and human studies on impulsivity and compulsivity used direct measures of impulsive and compulsive drug seeking behavior (91, 109). Additionally, cautious interpretation of the findings is warranted due to methodological effects. The applied questionnaires of addictive behaviors measure impulsive and compulsive symptoms and characteristics of these behaviors. This might contribute to inflated associations between measures of behavioral addictions and impulsivity and compulsivity among individuals with higher symptomatic severity. Alternatively, it might be possible that the increase in the level of correlations between impulsivity and compulsivity as a function of addictive behavioral symptom severity was presented as a result of a statistical artifact (e.g., those with higher addictive behavioral symptom severity had higher levels of impulsivity and compulsivity which contributed to greater variability and correlation between impulsivity and compulsivity). Future studies might consider controlling for these effects. The self-report measurements of behavioral addictions, impulsivity and compulsivity might also bias the findings. Finally, future studies might consider focusing only on those individuals who fulfill the diagnostic criteria of a given addictive disorder to have a more accurate view on how impulsivity and compulsivity are associated with problematic engagement in these behaviors.

## Conclusions

The current study shows that compulsivity dominates over impulsivity in the more severe symptomatic levels of behavioral addictions, possibly reflecting a shift from reward-driven to relief-driven and habitual behavior. On the theoretical level, our data supports theories that propose a transition from non-addictive to addictive behavior that occurs via a shift in *motivation* to pursue a given activity from being rewarding to avoiding unpleasant feelings. This shift has previously been described in animal models of addiction based on neuroscientific evidence. Our study supports these findings and strengthens the theory by providing evidence from a survey study with humans, although longitudinal data is needed for a full confirmation. We hope that these findings may enrich preventive efforts, psychological and pharmacological treatments, and diagnostic systems, as well as inspire further research in the field.

## Data Availability Statement

The raw data supporting the conclusions of this article will be made available by the authors, without undue reservation.

## Ethics Statement

The studies involving human participants were reviewed and approved by Institutional Review Board of Faculty of Education and Psychology, Eötvös Loránd University. The patients/participants provided their written informed consent to participate in this study.

## Author Contributions

ZD and BP designed the study and supervised data collection. WB provided expert counseling. AM analyzed the data. All authors participated in drafting and writing the manuscript and contributed to its revision. All authors take responsibility for the integrity of the data and approve the final version of the manuscript.

## Funding

This study was supported by the Hungarian National Research, Development and Innovation Office (Grant No. KKP126835, K128614, and K134807). ZH was supported by the ÚNKP-21-4 New National Excellence Program of the Ministry for Innovation and Technology from the source of the National Research, Development and Innovation Fund.

## Conflict of Interest

ELTE Eötvös Loránd University receives funding from the Szerencsejáték Ltd. to maintain a telephone helpline service for problematic gambling. ZD has also been involved in research on responsible gambling funded by Szerencsejáték Ltd. and the Gambling Supervision Board and provided educational materials for the Szerencsejáték Ltd's responsible gambling program. The remaining authors declare that the research was conducted in the absence of any commercial or financial relationships that could be construed as a potential conflict of interest.

## Publisher's Note

All claims expressed in this article are solely those of the authors and do not necessarily represent those of their affiliated organizations, or those of the publisher, the editors and the reviewers. Any product that may be evaluated in this article, or claim that may be made by its manufacturer, is not guaranteed or endorsed by the publisher.
