# Supplementary material for: Relating Compulsivity and Impulsivity With Severity of Behavioral Addictions: A Dynamic Interpretation of Large-Scale Cross-Sectional Findings
Source: Front Psychiatry. 2022 Jun 17;13:831992. doi: 10.3389/fpsyt.2022.831992 (PMC9248365; doi:10.3389/fpsyt.2022.831992)
Supplement: Supplementary file 1 [file Data_Sheet_1.docx]

**Appendix 1: Representative sample: distribution of the data and linear regression between impulsivity and compulsivity.**

#####


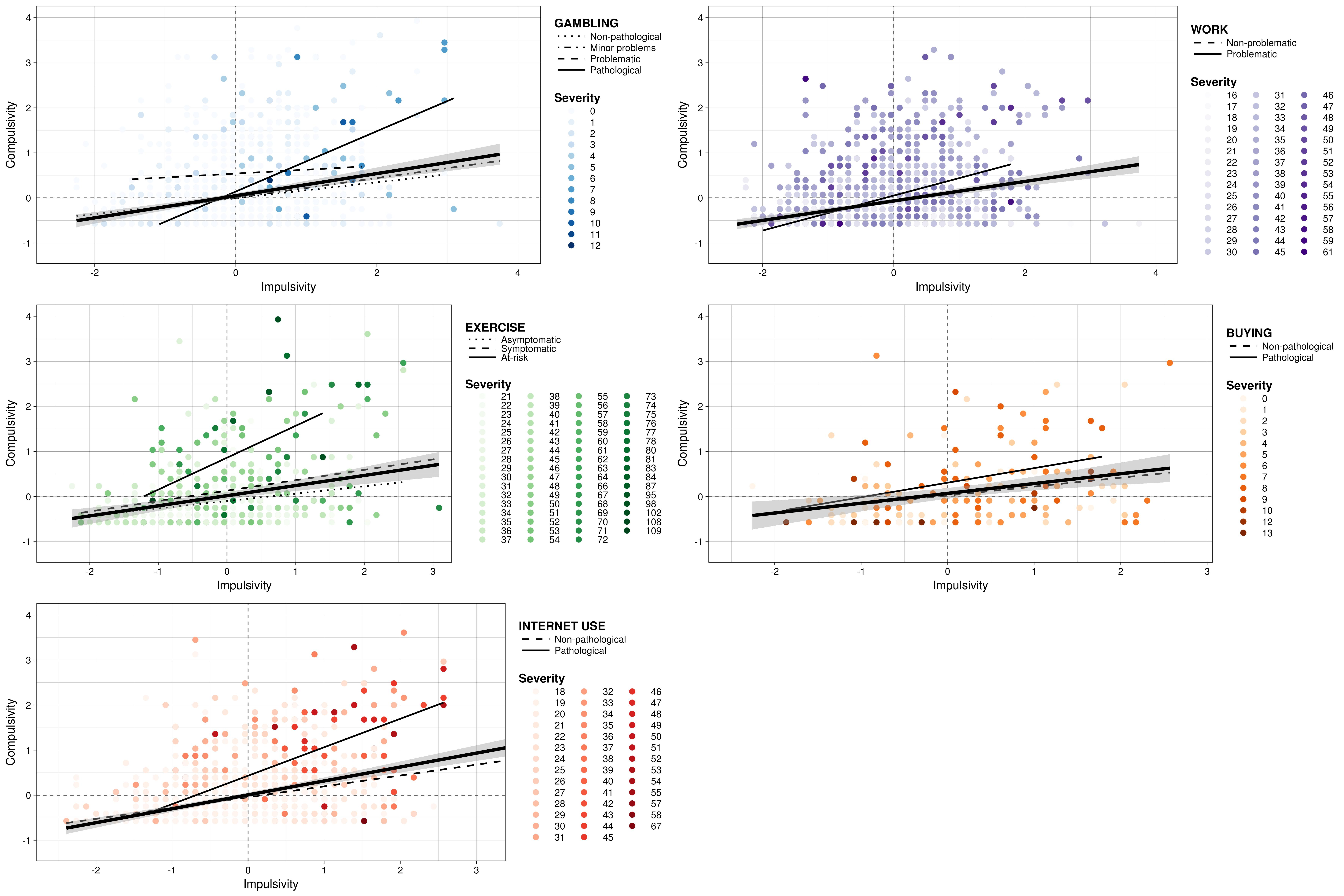


*Note: The linear regression line between impulsivity and compulsivity appears as a thick solid line with 95% confidence interval. Within-group regression lines appear as indicated on the legend. Impulsivity and compulsivity values were standardised within the sample, and severity score corresponds to the total score on the given instrument (see Methods)*

**Appendix 2:** **Specific samples: distribution of the data and linear regression between impulsivity and compulsivity.**

**
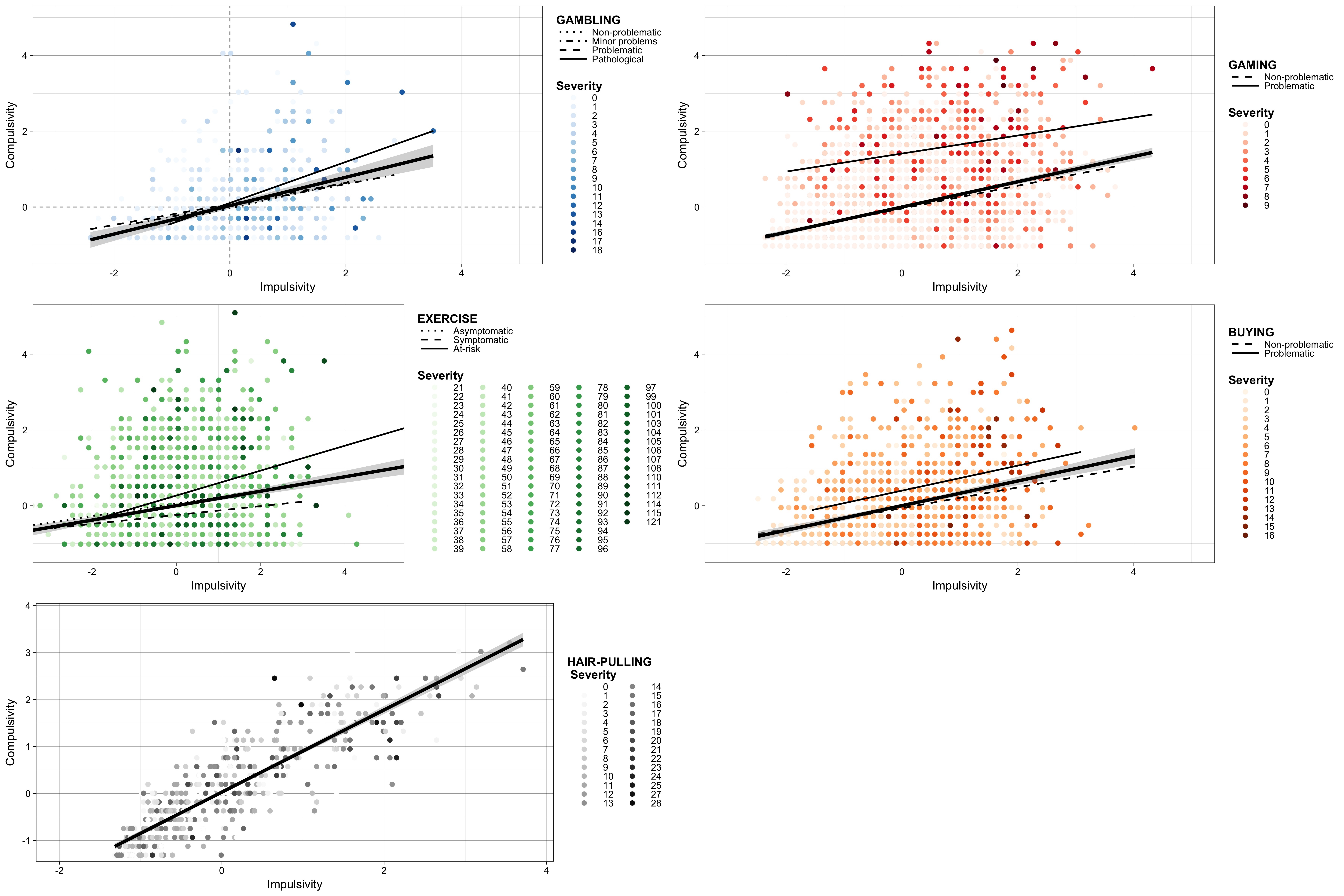
**

*Note: The linear regression line between impulsivity and compulsivity appears as a thick solid line with 95% confidence interval. Within-group regression lines appear as indicated on the legend. Impulsivity and compulsivity values were standardised within each sample, and the severity score corresponds to the total score on the given instrument (see Methods).*
